# Drying date plum (*Diospyros lotus* L.) fruit: Assessing rehydration properties, antioxidant activity, and phenolic compounds

**DOI:** 10.1111/1750-3841.16322

**Published:** 2022-09-16

**Authors:** Awadalgeed M. A. Hassan, Oscar Zannou, Hojjat Pashazadeh, Ali Ali Redha, Ilkay Koca

**Affiliations:** ^1^ Department of Food Engineering, Faculty of Engineering Ondokuz Mayis University Samsun Turkey; ^2^ Department of Public Health and Sport Sciences (Medical School) Faculty of Health and Life Sciences University of Exeter Exeter UK; ^3^ Centre for Nutrition and Food Sciences, Queensland Alliance for Agriculture and Food Innovation (QAAFI) The University of Queensland Brisbane Australia

**Keywords:** antioxidant activity, date plum, drying, phenolic compounds, rehydration

## Abstract

Date plum (*Diospyrus lotus* L.) is an edible fruit from the Ebenaceae family, rich in nutrients, and having tremendous medicinal properties. This paper attempted to show the influence of different parameters of convective drying such as temperature (50, 60, 70, and 80°C) and air velocity (0.5, 1.0, and 1.5 m/s) on the shrinkage and microstructure, rehydration properties, antioxidant activity, and phenolic compounds of date plum. The drying caused significant changes in the color, actual size, and distribution of the fruit cells of date plum. The total phenolic content (TPC), total flavonoid content (TFC), ferric reducing antioxidant power (FRAP), and 2,2‐diphenyl‐1‐picrylhydrazyl (DPPH) of fresh date plum were 0.81 ± 0.00 mg GAE/g, 0.23 ± 0.10 mg ECE/g, 7.15 ± 1.09 mmol ISE/g, and 14.92 ± 0.88 mmol/TE, respectively. The drying at 70°C had the highest values of TPC, TFC, gallic acid, chlorogenic and syringic acids, catechin, quercetin‐3‐glucoside, resveratrol, and DPPH. The drying air velocities showed no significant effects on the antioxidant contents and the antioxidant activity. Of the models applied to the drying kinetics, the Midilli model was found as the best model to describe the drying kinetics of date plum. In addition, the Weibull model was found as the most successful among the models applied to the rehydration kinetics of date plum. According to the achieved findings, the convective drying temperature of 70°C is the optimum temperature to produce the dehydrated date plum.

**Practical Application:**

This work has revealed the drying conditions responsible for preserving the phenolic compounds, total flavonoid content, and antioxidant features of *D. lotus* L. The study found the optimum drying conditions, and Midilli and Weibull models were the most fitted models to describe the drying and rehydration behaviors of *D. lotus* L. fruits, respectively. The drying provides a reasonable value of the possibility of continuous consumption of the fruits dried afforded on off‐seasons. The dried fruits are widely used for multipurpose and have been extensively used in food industries due to their rich nutraceutical and antioxidant compounds.

## 1 INTRODUCTION

Date plum or Caucasian persimmon (*D. lotus* L.) is from the Ebenaceae family and is native to China and Asia. Date plum is cultivated in several countries for its edible fruits, owing higher nutritive and medicinal properties. The consumption of fruits is recommended for their beneficial effects on human health including sedative, antiseptic, antidiabetic, antitumor, laxative, antidiarrhea, dry cough reliever, and tension regulator (Rashed et al., 2012; Uddin et al., 2011). Studies have reported the effectiveness of date plum of having antinociceptive (Uddin et al., 2014), muscle relaxative (Rauf et al., 2015), anti‐inflammatory (Uddin et al., 2014), antioxidant, antiproliferative (Loizzo et al., 2009; Nabavi et al., 2009), skin lesion recoverer (Azadbakht et al., 2011; Cho et al., 2017), sedative (Rauf et al., 2015; Uddin et al., 2014), and evidence anti‐HIV (Rashed et al., 2012) activities. The phytochemical studies of date plum revealed that it is a rich source of gallic acid, vanillic acid, caffeic acid, ferulic acid, salicylic acid, protocatechuic acid, myricetin, 3,4‐dihydroxybenzoic acid, quercetin, 4‐hydroxybenzoic acid, and ρ‐coumaric acid (Ayaz et al., 1997; H. Gao et al., 2014). Similarly, Rashed et al. (2012) have reported ellagic acid, methyl gallate, gallic acid, myricetin‐3‐O‐β‐glucuronide, myricetin‐3‐O‐α‐rhamnoside, myricetin, and quercetin as major in Egyptian date plum. These bioactive compounds have got potential application in the food, cosmetic, and pharmaceutical industries. Therefore, applying preservation techniques to have long‐term storage date plum with high functional properties is essential.

Although the date plum contains many essential bioactive compounds and has displayed many biological activities, it is a seasonal fruit with higher water content and is subjected naturally and rapidly to biochemical alterations. Thus, drying date plums seems opportune and timely to decrease the water content, limit microbiological and biological alterations, and preserve the nutritional and bioactive compounds as well as biological activities (A. Ahmad et al., 2012; Pashazadeh et al., 2020a, 2020b). Many drying techniques have been developed to preserve food quality and reduce environmental issues and energy consumption (Doymaz, 2008; Erbay & Icier, 2009; Figiel, 2010; Giri & Prasad, 2007). Convective drying is a hot air reported to be adequate for drying fruits and vegetables, providing faster, hygienic, and safe dried products (Pashazadeh et al., 2020a, 2020b; Zhu et al., 2020). The convective drying has many advantages that justify its wide use in the food industry. It corresponds to the basic treatment mode of drying food products for their conservation and gain benefits to prolonging the shelf life of the bioproducts such as fruits and vegetables (Kouhila et al., 2020). According to Almeida et al. (2016), the convective drying, compared to other types of drying has the advantages to be the low price of facilities, easy, and cheap process control as well as flexibility. It is a comparably economical drying technique with a well‐known theoretical framework (Kouhila et al., 2020). By a selection of a convenient convective mode of drying, the appropriate physical parameters such as temperature, humidity, and airflow rate, with a reasonable heat dosage are very essential for a better performance of drying (Almeida et al., 2016; Kouhila et al., 2020). Moreover, investigating the optimum drying conditions and kinetics became essential to evaluate the drying impacts on preserving bioactive compounds, nutritional quality, and antioxidant activity of the dried fruits (Pashazadeh et al., 2020a, 2020b).

The degree of rehydration is one of the most important factors determining the quality of the dried products. Usually, most dried agricultural products are rehydrated for their uses. The rehydration comprises three simultaneous processes: the imbibition of water into dried material, the swelling, and the leaching of solubles (Krokida & Marinos‐Kouris, 2003; Pashazadeh et al., 2020a). However, to date, no study has investigated the drying and rehydration conditions and kinetics of date plum. Thus, the main aim was to determine the drying conditions to have maximum total phenolic content (TPC), total flavonoid content (TFC), and the antioxidant activity of date plum. Furthermore, the kinetics and modelling of drying and rehydration as well as the microstructural changes occurring during the drying process were evaluated.

## 2 MATERIALS AND METHODS

### 2.1 Plant material

Date plum (*D. lotus* L.) fruits were collected from Samsun (Turkey) in 2019 during the Winter harvest season. Fruits were sorted, filled in PTE bags (ca. 300 g), and kept in the refrigerator.

### 2.2 Chemical and reagents

2,2‐Diphenyl‐1‐picrylhydrazyl (DPPH), 2,4,6‐tris(2‐pyridyl)−1,3,5‐triazine (TPTZ), acetone, Trolox, sodium nitrite, hydrochloric acid, methanol, sodium hydroxide, Folin‐Ciocalteau reagent, (−)‐ epicatechin, hesperidin, catechin, fumaric acid, and quercetin were purchased from Sigma‐Aldrich. Gallic acid (GA) and sodium carbonate were purchased from Riedel‐de Haen. Sodium acetate and glacial acetic acid were bought from Carlo Erba. Aluminum (III) chloride and iron (III) chloride were acquired from Merck.

### 2.3 Physicochemical characteristics

The moisture content was measured in an oven at 70°C for 24 h according to the preliminary tests. The color was determined using a colorimeter (DP‐400, Minolta, Japan) and the CIE L*a*b* scale was measured. The total soluble solids were evaluated at 25°C using an Abbe refractometer (Atago, Japan), and pH was measured with a pH meter.

### 2.4 Drying system and experiments

The drying system was a convective regime cabinet dryer (EKSIS, Turkey) described in our previous study (Pashazadeh et al., 2021, 2020a). The drying process was carried out at four temperatures (50, 60, 70, and 80°C) and three air velocities (0.5, 1.0, and 1.5 m/s). After the drying process, the samples were taken out to room temperature and sealed into polyethylene LDPE bags.

### 2.5 Modeling of drying curves

The moisture ratio (MR) was determined using Equation (3):

(1) $$MR=\frac{M_{t}}{M_{0}}\;$$

where *M*_0_ and *M_t_* are initial moisture and at time t, respectively.

The drying data was applied to six models (Table 1), and the coefficient of determination (*R* ^2^), chi‐square (χ^2^) and root mean square error (RMSE) were generated by MATLAB software (R2016d) to validate the fitness of the models (Doymaz, 2008; Zannou et al., 2021). The *R* ^2^, χ^2^, and RMSE were expressed as follows:

(2) $$R^{2}=1-\left(\frac{\sum_{i=1}^{N}{\left(MR_{p,i}-MR_{e,i}\right)}^{2}}{\sum_{\dot{I}=1}^{N}{\left(MR_{e,i}-{\bar{MR}}_{e,i}\right)}^{2}}\right)$$

(3) $$\chi^{2}=\frac{{\sum_{i=1}^{N}\left(MR_{e,i}-MR_{p,i}\right)}^{2}}{N-n}\;$$

(4) $$\mathrm{RMSE}={\left(\frac{1}{N}{\sum_{i=1}^{N}\left(MR_{e,i}-MR_{p,i}\right)}^{2}\right)}^{1/2}$$

where *MR_e_*_,i_ and *MR_p_*_,i_ are the *i*th experimental and predicted moisture rates, respectively. ${\bar{MR}}_{e,i}$ is the mean of the experimental moisture ratio. *N* and *n* are the number of treatments and the number of constants in the models, respectively.

**TABLE 1:** Empirical models applied to drying kinetics of date plum.

No:	Models	Equations	References
1	Newton	*MR* = exp (−*kt*)	Bengtston et al. (1998) et al. (1998); Tunde‐Akintunde (2011)
2	Page	*MR* = exp (−*kt^n^*)	Keneni et al. (2019)
3	Henderson and Pabis	*MR* = α exp (−*kt^n^*)	Özdemir & Devres, 1999; Yaldız et al. (2001)
4	Two‐term	*MR* = α exp (−*kt*) + *b* exp (−gt)	Nurafifah et al. (2018); Özdemir and Devres (1999)
5	Two‐term exponential	*MR* = α exp (−*kt*)+(1+α) exp (−*kαt*)	Chielle et al. (2016); Padoin et al. (2016)
6	Wang and Singh	*MR* = 1 + *at* + *bt* ^2^	Özdemir and Devres (1999); Nurafifah et al. (2018)
7	Approximation of diffusion	*MR* = α exp (−kt) + (1−α) exp (−*kbt*)	Ertekin and Yaldız (2004)
	Logistic	*MR* = α/(1 + *b* exp (*kt*))	Yaldız et al., 2001; Yaldız and Ertekin (2001); Samani et al. (2018)
8	Midilli	*MR* = α exp (−*kt^n^*) + *bt*	Nurafifah et al. (2018)
9	Aghabashlo model	*MR* = exp [−kt/(1+*gt*)]	Kumar et al. (2017)

*Note*: *MR* is the moisture ratio; t is the time; and α, *b*, *c*, g, *k*, and *n* are the constants of models; exp is the exponential function.

### 2.6 Sample extraction

A portion of 1 g of the crushed samples was mixed with 20 ml of 80% methanol and left to macerate for 12 h at 25°C. The mixtures were filtered and properly diluted for the analyses.

### 2.7 Total phenolic content

The TPC was determined by the Folin‐Ciocalteu method adopted from Zannou and Koca (2020). Briefly, 150 µl of the diluted sample was mixed 750 µl of 10% Folin‐Ciocalteu reagent (stood for 5 min) and 600 µl of 7.5% (w/v) Na_2_CO_3_. The mixture was placed in the dark for 2 h. The absorbance was read at 760 nm using a UV‐spectrophotometer (Thermo Spectronic) and expressed as mg gallic acid equivalent per g (mg GAE/g).

### 2.8 Total flavonoid content

The TFC was determined using the protocol of Zannou and Koca (2020). One milliliter of the diluted solution was combined with 0.3 ml of 5% NaNO_2_ and left to stand for 5 min, followed by the addition of 0.5 ml of 5% AlCl_3_. The mixture was kept for 6 min before adding 0.5 ml of 1 M NaOH. After 10 min, the absorbance was read at 510 nm. The TFC was estimated based on a calibration curve using epicatechin as standard. The results were given as mg epicatechin equivalents (ECE)/g dw.

### 2.9 DPPH radical scavenging activity

The DPPH radical scavenging was determined using the method described in Zannou and Koca (2020). Briefly, an aliquot of 50 µl sample was added with 1 ml DPPH solution (0.06 mM in 80% methanol). The mixture was shaken and left to stand in dark for 1 h until the reaction completed. Thereafter, the absorbance at 517 nm was recorded. The DPPH solution was used as control. The reduction ratio of DPPH was determined with the following equation:

(5) $$\mathrm{Reduction}\left(\%\right)=\left(\frac{A_{c}-A_{s}}{A_{c}}\right)\times 100$$

where *A_c_* is the absorbance of the control and *A_s_* is the absorbance of extract.

### 2.10 Ferric reducing antioxidant power

Ferric reducing antioxidant power (FRAP) assay was performed according to the procedure of Zannou and Koca (2020). Briefly, a portion if 50 ml volume of sample was mixed with 950 mM of FRAP solution constituted of 100 mM acetate buffer:10 mM FeCl_3_:10 mM TPTZ (2,4,6‐tripyridyl‐s‐triazine). The assembly was shaken for about 5 min, and the absorbance was read at 593 nm against a blank. The FRAP values of the extracts were calculated from the calibration curve using FeSO4 as a standard. The results were given as mmol FeSO_4_ equivalents (mmol ISE/g dw).

### 2.11 Rehydration curves and modeling

The rehydration was conducted at room temperature (25°C), and the rehydration features such as moisture content (*M_c_*) and rehydration ratio (*R_r_*) were calculated according to Pashazadeh, Zannou, Koca (2020). Peleg, Weibull, and Vegas‐Gálves models (Tables 2). were applied to the rehydration data, and the statistically significant results were shown based on *R* ^2^, χ^2^, and RMSE as expressed in Equations (2)–(4).

**TABLE 2:** Models applied to the rehydration kinetics of date plum.

Models	Equations	References
Peleg	$M\left(t\right)=M_{0}+\frac{t}{\left(\alpha +bt\right)}$	Vega‐Gálvez et al. (2009)
First‐order kinetic	*M*(*t*) = *Meq* + (*M*_0_−*Meq*) exp (−α *t*)	Benseddik et al. (2019), Ghellam and Koca (2020), and Krokida and Marinos‐Kouris (2003)
Exponential‐related equation	*M*(*t*) = *Meq* (1−exp (−α t))	Ghellam and Koca (2020) and Noshad et al. (2012)
Exponential model	*M*(*t*) = *Meq* + (*M*_0_−*Meq*) exp (−α tk)	Benseddik et al. (2019) and Saguy et al. (2005)
Weibull	*M*(t) = *Meq*+(*M*_0_−*Meq*) exp (−(*t*/*b*) α)	Benseddik et al. (2019), Pramiu et al. (2015), and Vega‐Gálvez et al. (2009)

*Note*: *M_t_*, *M*_0_, and *M_e_* are the water contents at time t, before rehydration, and end of rehydration, respectively, and α, b, and k are the constants of models.

### 2.12 LC‐MS/MS analysis

The phenolic compounds of fresh and dried samples were determined using liquid chromatography coupled to a mass spectrometer detector (LC‐MS/MS, Shimadzu LC‐MS 8040) as described in Zannou et al. (2021). The liquid chromatography coupled to a mass spectrometer detector (LC‐MS/MS, Shimadzu LC‐MS 8040) via electrospray ionization (ESI) and two pumps (LC‐30 AD), a column oven (CTO‐10AS VP), an autosampler (SIL‐30AC), and a degassing unit (DGU‐20A 3R). The MS/MS system functioned at 300°C capillary temperature, 350°C vaporizer temperature, 30 arb sheath gas pressure, 13 Arb Aux gas pressure, 4000 V spray voltage (positive polarity), 2500 V spray voltage (negative polarity), and 4 µA discharge current. A 0.45‐µm nylon filter was used to filter the samples and standards before injecting 20 µl into a C18 reversed‐phase column (ODS hypersil 5 µm, 4.6 × 250 mm). The column temperature was set at 30°C, and analysis was performed for 34 min. The mobile phase was constituted of water: formic acid in 99.9:0.1 v:v (mobile phase A) and HPLC grade methanol (mobile phase B). The flow rate of the solvents was 0.7 ml/min, and the following gradient solution was used: 0 min, 100% A; 1 min, 100% A; 22 min, 5% A and 95% B; 25 min, 5% A and 95% B; 30 min, and 0% A and 100% B. The phenolic compounds were identified based on their elution time and quantified from their peak area. The phenolic compounds were identified based on their elution time and quantified from their peak area. The identified compounds were quantified using a mixture of external standards (gallic acid, catechin, fumaric acid, and quercetin) prepared by dissolving standards in methanol at concentrations of 0, 50, 75, 100, 150, and 200 ppm.

### 2.13 Scanning electron microscope

The fresh and dried samples’ microstructures were obtained using scanning electron microscope (SEM) (JEOL JSM‐7001F) (Zannou et al., 2021).

### 2.14 Data analysis

The MATLAB software (R2016d) was used for the modelling and the Design‐Expert software 9.0 (Trial version, Stat‐Ease Inc., Minneapolis, USA) to generate the optimization models and one‐factor graphics. The statistical significance of independent variables and the correlation between them was evaluated using ANOVA. The adequacy of the optimization model was determined based on the coefficient of determination (R^2^), adjusted coefficient of determination (adj. R^2^), coefficient of variation (CV), and Fisher's test value (F‐value). The regression coefficients were considered significant at p < 0.05. The optimum parameters were estimated considering the desirability function. The analyses were carried out in triplicate, and the one‐way ANOVA with post hoc Duncan's test was used (SPSS, version 21). The significance of the results was given at p≤ 0.05.

## 3 RESULTS AND DISCUSSION

### 3.1 Characteristics of raw material

The date plum studied in the present work is almost spherical (19.90 mm in width and 18.06 mm in length) with a flesh/seed ratio of 2.81 and the number of grains is about 220 for each kilogram. As shown in Table 3, the dry matter, soluble solids, and pH were 44.60%, 13.66%, and 6.82, respectively. It is encouraging to compare soluble solids and dry matter results with that found by Ayoub et al. (2020), who found that total soluble solids and moisture of date plum pulps are 13% and 70.5%, respectively. The color of the date plum was determined as L*, a*, and b* values were 37.20, 6.40, and 14.48, respectively.

**TABLE 3:** Physicochemical characteristics of fresh date plum.

Fresh fruit characteristics	Values^a^
Number of grains per kg	220 ± 10
Width, mm	19.90 ± 5.03
Length, mm	18.06 ± 2.75
Flesh/seed ratio	2.81 ± 0.95
Dry matter, %	44.60 ± 1.99
Soluble solids, %	13.66 ± 1.96
pH	6.82 ± 0.17
TPC, mg GAE/g	0.81 ± 0.01
TFC, mg ECE/g	0.23 ± 0.10
FRAP, mmol ISE/g	7.15 ± 1.09
DPPH, mmol TE/g	14.92 ± 0.88
Color features	
L*	37.20 ± 3.86
a*	6.40 ± 2.49
b*	14.48 ± 3.90

^a^ Means values of three replicates and corresponding standard deviations.

In this study, the TPC, TFC, and antioxidant activities (FRAP and DPPH) were determined (Table 1). Evidence suggested that the potent medicinal properties agents of date plum could be correlated with various bioactive compounds (Uddin et al., 2011). The present results showed that the FRAP and DPPH values of date plum extracts were 7.15 ± 1.09 mmol ISE/g and 14.92 ± 0.88 mmol TE/g, respectively. The TPC and TFC were determined as 0.81 ± 0.01 mg GAE/g and 0.23 ± 0.10 mg ECE/g, respectively. There are similarities between our findings in this study and those described by Murathan (2020), who found that TPC was 1.3 mg/g and TFC was 0.12 mg/g. It has previously been observed that the flavonoid content of date plum ranged from 30.52% to 34.42%, and the phenol content ranged from 16.05% to 17.40% (M. Ahmad et al., 2014).

### 3.2 Modeling of drying kinetics

**FIGURE 1:**
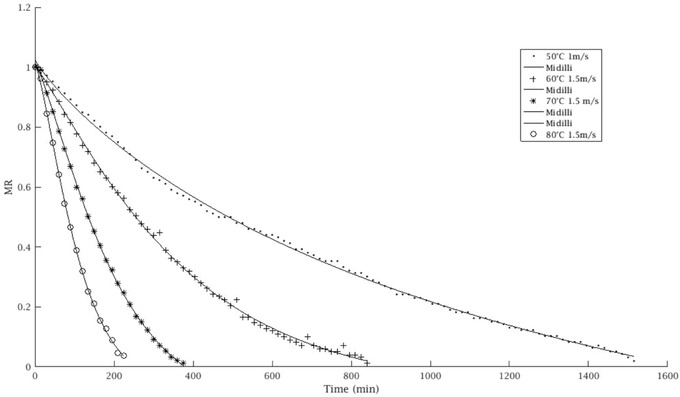
Drying curves of *Diospyros lotus* L fruits.

The results of modeling applied to the drying kinetics of date plum are shown in Table 2. As it can be observed, an increase in temperature resulted in an important reduction in drying time (Figure 1). The fruit dried at 50°C showed the longest drying time, and the drying time decreased as the temperature increased. These results were in agreement with those obtained by Koca et al. (2009) who reported that the drying time decreased as the airflow rate and temperature increased. They also found the drying time increased as the temperature decreased. The drying curves were applied to Newton, Page, Henderson and Pabis’, Two‐term, Two‐term exponential, Logistic, Midilli, and Aghabashlo models to find out the well‐fitted models describing the dehydration behavior. This approach used in this investigation is similar to that used by (Pashazadeh, Zannou, & Koca, 2020; Zannou et al., 2021). Furthermore, as shown in Figure 1, the results of (*MR*, t) obtained for the different temperatures indicated that the Midilli model was the well fitted with the experimental data giving the highest coefficient of determination (*R* ^2^), the lowest RMSE and lowest chi‐square (χ^2^) (Table 4). These results were found similar to those of the drying kinetics of *R. pimpinellifolia* (Pashazadeh et al., 2020a). In the Midilli model at 50, 60, 70, and 80°C at air velocity 0.5, 1.0, and 1.5 m/s, the results of *R* ^2^ varying from 0.9968251 to 0.9995381, RMSE from 0.0165964 to 0.0074116, and χ^2^ from 0.0002754 to 0.000054. Midilli model was the best fitted at the drying conditions of 70°C and 1.5 m/s, giving *R* ^2^ of 0.9995381, RMSE of 0.0074116, and χ^2^ of 0.000054.

**TABLE 4:** Mathematical models applied to experimental drying kinetics of date plum.

Model	Drying temperature (°C)	Air velocity, m/s	*R* ^2^	RMSE	χ^2^	Constants
**Newton**	50	0.5	0.981368	0.0396265	0.0015702	k = 0.0017103
		1.0	0.989209	0.0281931	0.0007948	k = 0.0015122
		1.5	0.896501	0.1036942	0.0107525	k = 0.0022623
	60	0.5	0.977901	0.0437773	0.0019164	k = 0.0032194
		1.0	0.983023	0.0377495	0.0014250	k = 0.0030109
		1.5	0.980623	0.0412749	0.0017036	k = 0.0030194
	70	0.5	0.953837	0.0684128	0.0046803	k = 0.0046568
		1.0	0.958493	0.0658683	0.0043386	k = 0.0061028
		1.5	0.955910	0.0679314	0.0046146	k = 0.0059595
	80	0.5	0.947262	0.0722411	0.0052187	k = 0.0059928
		1.0	0.963218	0.0626485	0.0039248	k = 0.0086279
		1.5	0.960261	0.0655185	0.0042926	k = 0.0094659
**Page**	50	0.5	0.996181	0.0180266	0.0003249	k = 0.0003604
						n = 1.2393
		1.0	0.993688	0.0216710	0.0004696	k = 0.000711
						n = 1.1146
		1.5	0.9847422	0.0401946	0.0016156	k = 1.5625
						n = 1.8188
	60	0.5	0.9944488	0.0221686	0.0004914	k = 0.0007454
						n = 1.2506
		1.0	0.9946105	0.0214773	0.0004612	k = 0.000924
						n = 1.2001
		1.5	0.9975685	0.0147533	0.0002176	k = 0.0006554
						n = 1.2579
	70	0.5	0.9964337	0.0193294	0.0003736	k = 0.0003624
						n = 1.4701
		1.0	0.9970025	0.0180513	0.0003258	k = 0.000578
						n = 1.453
		1.5	0.9971543	0.0176139	0.0003102	k = 0.0005283
						n = 1.4656
	80	0.5	0.9971949	0.0170936	0.0002921	k = 0.0004347
						n = 1.5158
		1.0	0.9966150	0.0195563	0.0003824	k = 0.0011696
						n = 1.4128
		1.5	0.9987755	0.0119047	0.0001417	k = 0.0011264
						n = 1.4514
**Hendeon vs. Pabis**	50	0.5	0.9873884	0.0327583	0.0010731	a = 1.0733
						k = 0.0018356
		1.0	0.9909311	0.0259757	0.0006747	a = 1.0345
						k = 0.0015688
		1.5	0.9247374	0.0892714	0.0079693	a = 1.1484
						k = 0.0026284
	60	0.5	0.9849675	0.0364804	0.0013308	a = 1.0746
						k = 0.003468
		1.0	0.9894251	0.0300846	0.0009050	a = 1.0702
						k = 0.0032332
		1.5	0.9884351	0.0321757	0.0010352	a = 1.0815
						k = 0.0032685
	70	0.5	0.9727583	0.0534230	0.0028540	a = 1.1244
						k = 0.0052522
		1.0	0.9737137	0.0534564	0.0028575	a = 1.1145
						k = 0.0067923
						g = 0.012407
						k = 0.011737
						a = 1.1196
		1.5	0.9732444	0.0540097	0.0029170	k = 0.0066786
	80	0.5	0.9695994	0.0562732	0.0031666	a = 1.122
						k = 0.00681
		1.0	0.9768348	0.0511597	0.0026173	a = 1.1046
						k = 0.0095281
		1.5	0.9753737	0.0533877	0.0028502	a = 1.1055
						k = 0.010492
**Two term**	50	0.5	0.9962413	0.0179598	0.0003225	a = −23.472
						b = 24.4828
						g = 0.0028147
						k = 0.0028864
		1.0	0.9939897	0.0211464	0.0004471	a = 1.6022
						k = 0.0019137
						k = 0.0011247
		1.5	0.9704335	0.0570610	0.0032559	a = −25.5853
						b = 26.606
						g = 0.0049774
						k = 0.0051809
	60	0.5	0.9865846	0.0352037	0.0012393	a = 1.0922
						b = −0.092202
						g = 2.0202
						k = 0.0035271
		1.0	0.9945081	0.0221184	0.0004892	a = 10.1482
						b = −9.1271
						g = 0.0050411
						k = 0.0047642
		1.5	0.9960761	0.0190924	0.0003645	a = 18.0476
						b = −17.0042
						g = 0.0050348
						k = 0.0048794
	70	0.5	0.9795309	0.0479338	0.0022976	a = 1.1716
						b = −0.17158
						g = 2.1855
						k = 0.0054782
		1.0	0.9952665	0.0236498	0.0005593	a = 16.2909
						b = −15.2832
						g = 0.012407
						k = 0.011737
		1.5	0.9814357	0.0469891	0.0022079	a = 1.1799
						b = −0.17993
						g = 1.4036
						k = 0.0070421
	80	0.5	0.9956269	0.0225634	0.0005091	a = −10.8136
						b = 11.8299
						g = 0.012334
						k = 0.013409
		1.0	0.9939033	0.0279404	0.0007806	a = −85.5128
						b = 86.5554
						g = 0.015664
						k = 0.015795
		1.5	0.9885335	0.0393488	0.0015483	a = 1.2045
						b = −0.20446
						g = 1.3864
						k = 0.011452
**Two‐term exponential**	50	0.5	0.9813502	0.0398358	0.0015868	a = 0.0001079
						k = 15.833
		1.0	0.9939897	0.0211464	0.0004471	a = 1.6021
						k = 0.0019135
		1.5	0.9678549	0.0583418	0.0034037	a = 2.0847
						k = 0.0038087
	60	0.5	0.9938541	0.0233257	0.0005440	a = 1.7883
						k = 0.0045222
		1.0	0.9941247	0.0224244	0.0005028	a = 1.7307
						k = 0.0040986
		1.5	0.9970141	0.0163491	0.0002672	a = 1.8005
						k = 0.0042533
	70	0.5	0.9538163	0.0695593	0.0048385	a = 6.7219
						k = 69.2663
		1.0	0.9942688	0.0249606	0.0006230	a = 1.9669
						k = 0.0094004
		1.5	0.9941453	0.0252648	0.0006383	a = 1.9768
						k = 0.0092801
	80	0.5	0.9472488	0.0741271	0.0054948	a = 3.561
						k = 168.2749
		1.0	0.9632011	0.0644804	0.0041577	a = 6.7217
						k = 128.3327
		1.5	0.9602085	0.0678637	0.0046054	a = 0.0001838
						k = 51.4765
**Wang vs. Singh**	50	0.5	0.9962787	0.0177943	0.0003166	a = −0.0012899
						b = 4.35827
		1.0	0.9930802	0.0226902	0.0005148	a = −0.0011687
						b = 3.66697
		1.5	0.9827875	0.0426917	0.0018225	a = −0.0013805
						b = 7.72998
	60	0.5	0.9965325	0.0175207	0.0003069	a = −0.0024063
						b = 1.48496
		1.0	0.9955790	0.0194520	0.0003783	a = −0.0022756
						b = 1.35046
		1.5	0.9985556	0.0113708	0.0001292	a = −0.002271
						b = 1.34136
	70	0.5	0.9938465	0.0253905	0.0006446	a = −0.0033461
						b = 2.57616
		1.0	0.9948559	0.0236475	0.0005592	a = −0.0044437
						b = 4.8176
		1.5	0.9964650	0.0196316	0.0003854	a = −0.0042538
						b = 4.17596
	80	0.5	0.9921204	0.0286491	0.0008207	a = −0.0041916
						b = 3.24356
		1.0	0.9931008	0.0279195	0.0007795	a = −0.0063349
						b = 9.92096
		1.5	0.9945827	0.0250398	0.0006269	a = −0.0068896
						b = 1.1219
**Approximation of diffusion**	50	0.5	0.9939385	0.0229319	0.0005258	a = 1.1481
						b = 0.28388
						k = 0.0012933
						t = 0.68994
		1.0	0.9976263	0.0134242	0.0001802	a = 1.1481
						b = 0.079938
						k = 0.0011625
						t = 0.75166
		1.5	0.9843555	0.0415069	0.0017228	a = 5.5398
						b = 4.8603
						k = 0.0002715
						t = 4.8873
	60	0.5	0.9968036	0.0171835	0.0002952	a = 1.2557
						b = 0.32915
						k = 0.0021166
						t = 0.46508
		1.0	0.9973144	0.0154672	0.0002392	a = 1.2129
						b = 0.16192
						k = 0.0020983
						t = −0.26934
		1.5	0.9967076	0.0174886	0.0003058	a = 1.1982
						b = 0.51316
						k = 0.0021289
						t = 0.51046
	70	0.5	0.9922183	0.0295549	0.0008734	a = 1.5962
						b = 1.07
						k = 0.002183
						t = 1.3554
		1.0	0.9913359	0.0319964	0.0010237	a = 1.41
						b = −0.017547
						k = 0.0033407
						t = 0.54081
		1.5	0.9949093	0.0246062	0.0006054	a = 1.5651
						b = 0.98357
						k = 0.002848
						t = 1.2507
	80	0.5	0.9953948	0.0231544	0.0005361	a = 1.7546
						b = 5.0548
						k = 0.0028761
						t = 5.0522
		1.0	0.9907122	0.0344861	0.0011892	a = 1.374
						b = 0.47945
						k = 0.0049329
						t = 0.91542
		1.5	0.9929367	0.0308830	0.0009537	a = 1.5158
						b = 1.1072
						k = 0.004788
						t = 1.0918
**Logistic**	50	0.5	0.9972175	0.0154614	0.0002390	a = 0.64501
						b = 1.6216
						k = 0.0027349
		1.0	0.9945248	0.0202848	0.0004114	a = 1.3609
						b = 2.325
						k = 0.0020193
		1.5	0.9913300	0.0305947	0.0009360	a = 0.074276
						b = 1.0255
						k = 0.0071504
	60	0.5	0.9950770	0.0210975	0.0004451	a = 0.67543
						b = 1.6663
						k = 0.0051582
		1.0	0.9947958	0.0213149	0.0004543	a = 1.0758
						b = 2.0978
						k = 0.0043452
		1.5	0.9980795	0.0132325	0.0001751	a = 0.68312
						b = 1.6822
						k = 0.0048235
	70	0.5	0.9967460	0.0187791	0.0003526	a = 0.32046
						b = 1.3326
						k = 0.009528
		1.0	0.9975146	0.0167762	0.0002814	a = 0.31711
						b = 1.318
						k = 0.012278
		1.5	0.9973940	0.0172184	0.0002964	a = 0.32245
						b = 1.332
						k = 0.012071
	80	0.5	0.9971574	0.0176788	0.0003125	a = 0.29696
						b = 1.3206
						k = 0.013054
		1.0	0.9961558	0.0214821	0.0004614	a = 0.41778
						b = 1.4401
						k = 0.01624
		1.5	0.9984013	0.0141159	0.0001992	a = 0.36085
						b = 1.3815
						k = 0.018802
**Midilli**	50	0.5	0.9968251	0.0165964	0.0002754	a = 0.97471
						b = −7.6114e‐06
						k = 0.0002867
						n = 1.2668
		1.0	0.9985253	0.0105810	0.0001119	a = 1.0265
						b = 0.0001078
						k = 0.0029099
						n = 0.86537
		1.5	0.9907348	0.0319422	0.0010203	a = 0.95364
						b = −9.4683
						k = 8.94356
						n = 1.8744
	60	0.5	0.9973938	0.0155162	0.0002407	a = 1.0094
						b = −0.000142
						k = 0.001765
						n = 1.075
		1.0	0.9980940	0.0130302	0.0001697	a = 1.0332
						b = −0.0001585
						k = 0.0030256
						n = 0.97142
		1.5	0.9986268	0.0112942	0.0001275	a = 1.0022
						b = −5.81835
						k = 0.0010282
						n = 1.1682
	70	0.5	0.9978387	0.0155755	0.0002425	a = 1.009
						b = −0.000154
						k = 0.0007054
						n = 1.3265
		1.0	0.9980341	0.0152410	0.0002322	a = 0.99694
						b = −0.00011378
						k = 0.000805
						n = 1.3733
		1.5	0.9995381	0.0074116	0.000054	a = 1.0121
						b = −0.0002408
						k = 0.0011829
						n = 1.2831
	80	0.5	0.9981349	0.0147351	0.0002171	a = 1.0149
						b = −0.0002550
						k = 0.0008396
						n = 1.3629
		1.0	0.9971854	0.0189842	0.0003604	a = 1.0103
						b −0.00014253
						k = 0.0017498
						n = 1.3178
		1.5	0.9994114	0.0089146	0.000079	a = 1.0108
						b = −0.0002027
						k = 0.0017355
						n = 1.345
**Aghabashlo model**	50	0.5	0.9965496	0.0171344	0.0002935	g = 0.00029098
						k = 0.0013299
		1.0	0.9960815	0.0170746	0.0002915	g = 0.00018775
						k = 0.00128
		1.5	0.9907674	0.0312668	0.0009776	g = −0.0010345
						k = 0.0011353
	60	0.5	0.9966765	0.0171530	0.0002942	g 0.00063324
						k = 0.002422
		1.0	0.9964123	0.0175232	0.0003070	g = 0.0005108
						k = 0.0023698
		1.5	0.9985907	0.0112320	0.0001261	g = −0.000572
						k = 0.0022829
	70	0.5	0.9961928	0.0199715	0.0003988	g = −0.0013991
						k = 0.0029749
		1.0	0.9973775	0.0168843	0.0002850	g = –0.001741
						k = 0.0039225
		1.5	0.9982474	0.0138230	0.0001910	g = 0.0017752
						k = 0.0037793
	80	0.5	0.9949600	0.0229125	0.0005249	g = −0.0020746
						k = 0.0037337
		1.0	0.9942199	0.0255549	0.0006530	g = −0.0022188
						k = 0.0058633
		1.5	0.9966071	0.0198163	0.0003926	g = −0.0027227
						k = 0.006204

Abbreviations: *R* ^2^, coefficient of determination; χ^2^, Chi square; RMSE, root mean square error.

### 3.3 Effects of drying on rehydration kinetics

**FIGURE 2:**
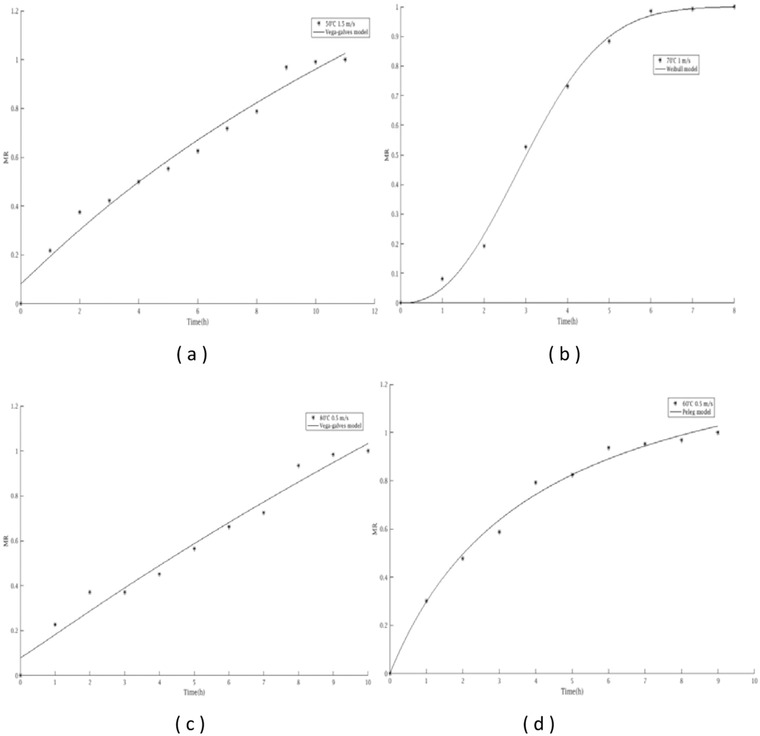
Rehydration curves changes in moisture content of dried samples for different temperatures of 50°C (a), 70°C (b), 80°C (c), and 60°C (d).

The rehydration ratio is one of the quality parameters of the dried products (Sharma et al., 2005). Experimental rehydration was performed on the dried date plum to evaluate the moisture uptake. The findings obtained after the rehydration different temperatures indicated that the drying temperature affected the behavior of the dried fruit. Similar to our findings, Chenlo et al. (2018) who reported that the rehydration capacity and water uptake permeability are associated with the increase in drying temperature. This phenomenon could be attributed to some substances being dragged by water flow during drying. Table 4 shows the rehydration parameters of dried fruit. The empirical models such as Peleg, first‐order kinetic, Exponential‐related equation, exponential, and Weibull were considered in this work. The dried samples showed fast rehydration in the first hours (5 h), followed by slower water absorption which achieved equilibrium after 6 h. As Figure 2, the rehydration is faster at 70°C, followed by 60, 80, and 50°C, respectively. Also, the Peleg, Vega‐Gálvez, and Weibull models depicted better rehydration behavior. These outcomes corroborate with various studies which mentioned that these models were adequate to the rehydration kinetics of various fruits (Benseddik et al., 2019; Pramiu et al., 2015; Vega‐Gálvez et al., 2009). Weibull model fit the experimental data well with *R* ^2^ higher than 0.99, lower χ^2^ (0.0005), and lower RMSE (0.0243) (Table 5).

**TABLE 5:** Results of the mathematical models for rehydration kinetics.

Model	Drying temperature (°C)	Air velocity, m/s	*R* ^2^	RMSE	χ^2^	Constants
**Peleg**	50	0.5	0.9686929	0.0667288	0.0044527	a = 17.3486
						b = −0.65285
		1.0	0.9728760	0.0616083	0.0037955	a = 7.2852
						b = 0.31459
		1.5	0.9732346	0.0542582	0.0029439	a = 6.1211
						b = 0.43799
	60	0.5	0.9913442	0.0331405	0.0010982	a = 2.6859
						b = 0.67551
		1.0	0.9613429	0.0752573	0.0056636	a = 5.3121
						b = 0.44827
		1.5	0.9284872	0.0845211	0.0071438	a = 4.6841
						b = 0.58621
	70	0.5	0.9657222	0.0645664	0.0041688	a = 4.1571
						b = 0.5218
		1.0	0.9445493	0.1038668	0.0107883	a = 4.9321
						b = 0.28925
		1.5	0.9323755	0.0892904	0.0079727	a = 5.6554
						b = 0.34262
	80	0.5	0.9692896	0.0601125	0.0036135	a = 6.8437
						b = 0.29861
		1.0	0.9518885	0.0681313	0.0046418	a = 1.7346
						b = 0.85804
		1.5	0.9386015	0.0853826	0.0072902	a = 7.1537
						b = 0.13063
**First‐order kinetic**	50	0.5	0.7929317	0.1636260	0.0267734	a = 0.13068
		1.0	0.9126146	0.105874	0.011209	a = 0.19639
		1.5	0.9402696	0.0772823	0.0059725	a = 0.20391
	60	0.5	0.9865899	0.0388906	0.0015124	a = 0.35809
		1.0	0.9264738	0.0989598	0.0097930	a = 0.23947
		1.5	0.9058394	0.0924723	0.0085511	a = 0.22575
	70	0.5	0.9410786	0.0798105	0.0063697	a = 0.27817
		1.0	0.8824753	0.1414464	0.0200071	a = 0.29788
		1.5	0.9008602	0.1011302	0.0102273	a = 0.23437
	80	0.5	0.9196772	0.0922280	0.0085060	a = 0.20637
		1.0	0.9218901	0.0823563	0.0067825	a = 0.37678
		1.5	0.8834898	0.1100213	0.0121046	a = 0.21805
**Exponential‐related equation**	50	0.5	0.7929317	0.1636260	0.0267734	a = 0.13068
		1.0	0.9126146	0.105874	0.011209	a = 0.19639
		1.5	0.9402696	0.0772823	0.0059725	a = 0.20392
	60	0.5	0.9865899	0.0388906	0.0015124	a = 0.35809
		1.0	0.9264738	0.0989598	0.0097930	a = 0.23947
		1.5	0.9058394	0.0924723	0.0085511	a = 0.22575
	70	0.5	0.9410786	0.0798105	0.0063697	a = 0.27818
		1.0	0.8824753	0.1414464	0.0200071	a = 0.29788
		1.5	0.9008602	0.1011302	0.0102273	a = 0.23437
	80	0.5	0.9196772	0.0922280	0.0085060	a = 0.20636
		1.0	0.9218901	0.0823563	0.0067825	a = 0.37677
		1.5	0.8834898	0.1100213	0.0121046	a = 0.21805
**Exponential model**		0.5	0.9630622	0.0724815	0.0052535	a = 0.0040572
						k = 2.7812
		1.0	0.974159	0.060134	0.003616	a = 0.052923
						k = 1.7389
		1.5	0.9462393	0.0768973	0.0059131	a = 0.15479
						k = 1.1588
	60	0.5	0.9909628	0.0338627	0.0011466	a = 0.29713
						k = 1.1489
		1.0	0.9829703	0.0499501	0.0024950	a = 0.075472
						k = 1.7362
		1.5	0.9069525	0.0964108	0.0092950	a = 0.25092
						k = 0.93778
	70	0.5	0.9457065	0.0812594	0.0066031	a = 0.2224
						k = 1.151
		1.0	0.9969621	0.0243113	0.0005910	a = 0.050986
						k = 2.3637
		1.5	0.9046163	0.1060449	0.0112455	a = 0.19404
						k = 1.1253
	80	0.5	0.9354478	0.0871520	0.0075954	a = 0.12642
						k = 1.2893
		1.0	0.9539740	0.0666383	0.0044406	a = 0.55556
						k = 0.70384
		1.5	0.8957626	0.1112506	0.0123767	a = 0.14683
						k = 1.2549
**Weibull**	50	0.5	0.9630622	0.0724815	0.0052535	a = 2.7802
						b = 7.2437
		1.0	0.9741590	0.060134	0.003616	a = 1.7386
						b = 5.4198
		1.5	0.9462393	0.0768973	0.0059131	a = 1.1587
						b = 5.0025
	60	0.5	0.9909628	0.0338627	0.0011466	a = 1.149
						b = 2.8755
		1.0	0.9829703	0.0499501	0.0024950	a = 1.7361
						b = 4.4295
		1.5	0.9069525	0.0964108	0.0092950	a = 0.93764
						b = 4.3674
	70	0.5	0.9457064	0.0812595	0.0066031	a = 1.1507
						b = 3.6909
		1.0	0.9969621	0.0243113	0.0005910	a = 2.3638
						b = 3.5223
		1.5	0.9046163	0.1060449	0.0112455	a = 1.1253
						b = 4.2936
	80	0.5	0.9354478	0.0871520	0.0075954	a = 1.2891
						b = 4.9733
		1.0	0.9539740	0.0666383	0.0044406	a = 0.70375
						b = 2.3051
		1.5	0.8957626	0.1112506	0.0123767	a = 1.2545
						b = 4.6127
**Proposed model**	50	0.5	0.9676514	0.0714987	0.0051120	a = 30,355,130.031
						b = 21.0753
						k = 0.081862
		1.0	0.9760471	0.060721	0.003687	a = 2.4604
						b = 5.42
						k = 0.731
		1.5	0.9771205	0.0528786	0.002796	a = 19.2216
						b = 5.4711
						k = 0.25114
	60	0.5	0.9908628	0.0364004	0.0013249	a = 1.2596
						b = 3.6482
						k = 1.2225
		1.0	0.9751682	0.0635794	0.0040423	a = 1.3496
						b = 7.4978
						k = 1.3517
		1.5	0.9473324	0.0764582	0.0058458	a = 51.3675
						b = 5.9818
						k = 0.17081
	70	0.5	0.9666538	0.0680799	0.0046348	a = 3.3499
						b = 3.747
						k = 0.50151
		1.0	0.9939106	0.0371776	0.0013821	a = 1.0957
						b = 43.4438
						k = 2.9432
		1.5	0.9359955	0.0938278	0.0088036	a = 303.3513
						b = 8.1708
						k = 0.16057
	80	0.5	0.9738120	0.0588775	0.0034665	a = 316.7003
						b = 8.3097
						k = 0.15532
		1.0	0.9409324	0.0800706	0.0064113	a = 1.2269
						b = 2.3532
						k = 0.98937
		1.5	0.9458233	0.0866304	0.0075048	a = 12,722.084
						b = 12.0156
						k = 0.10785

Abbreviation: RMSE, root mean square error.

### 3.4 Effects of drying on microstructure

**FIGURE 3:**
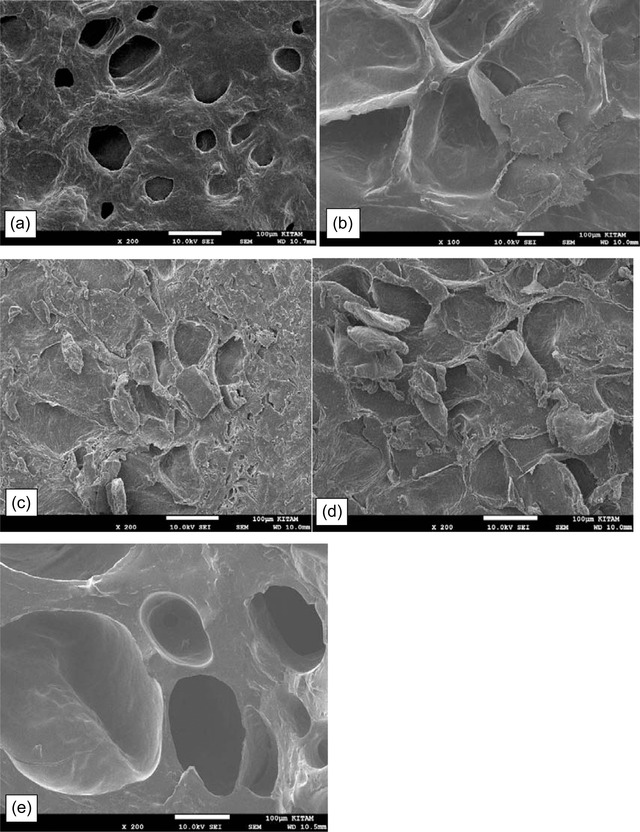
Microstructures (scanning electron microscopy) of the fresh sample (a) and dried *Diospyros lotus* L. fruits, (b) Fruits dehydrated at 50°C, (c) sample dehydrated at 60°C, (d) fruit dehydrated at 70°C, (e) fruits dehydrated at 80°C; all micrographs were taken on the surface of the sample.

The effects of drying on microstructure and distribution of cells in fresh and dried apples are presented in Figure 3. The SEM micrographs on the surfaces of raw and dried date plum fruits showed a distinct difference in the microstructure of fruits (Figure 3). Accordingly, Witrowa‐Rajchert and Rząca (2009) determined that the drying causes many changes in the structure and properties of plant material. The fresh date plum fruit tissue showed a well‐organized structure consisting of small and clear spherical to oval cells and intercellular spaces. The fruit dried at 50°C has large undistinguished cells spaces with the fruit dried at 60°C and 70°C showed a decreased intercell contact and collapse of cell structure up to the breakdown of cell walls. The SEM micrographs of fruit dried at 80°C have organized large cells, intercell contact, and well structure (Figure 3). Seemingly, Witrowa‐Rajchert and Rząca (2009) reported the drying of apples causes significant changes in the size of the cells and their distribution. In a previous study, the tissue of the fresh autumn olives showed large spherical and organized cell walls, while dried convective berries cells became very large, deformed, irregular, and wrinkled (Zannou et al., 2021). The results of the microstructure showed significant differences between fresh and dried date plum structural characteristics as well as significant changes in the cell size and distribution (Figure 3) Table 5.

### 3.5 Effects of drying on antioxidant properties

The values of TPC, TFC, FRAP, and DPPH of dried date plum are shown in Table 6. As can be seen, the TPC, TFC, and DPPH of dried fruit increased with increasing the temperature from 50°C to 70°C, while the FRAP did not vary irrespectively of the increase of temperature. The lower TPC, TFC, and antioxidant activity obtained at 50°C is due to the longer exposure of fruit which occurs in the degradation of the compounds with antioxidant activity (Koca et al., 2009; Pashazadeh et al., 2020a). The drying at 70°C gave the highest TPC, TFC, and DPPH of 4.44 mg GAE/g, 1.66 mg ECE/g, and 54.03 mmol TE/g, respectively. The drying at 80°C led to a decrease in TPC (from 4.44 mg GAE/g to 3.96 mg GAE/g), TFC (from 1.66 mg ECE/g to 0.79 mg ECE/g), and DPPH (from 54.03 mmol TE/g to 32.54 mmol TE/g). The increase of the drying temperature above 80°C induced the destruction of antioxidant substances in date plum. Similarly, it has been reported in previous studies that the drying agricultural material at higher temperatures (above 70°C) is harmful to antioxidants compounds, leading to the destruction of the structure of some phenolic compounds (Erbay & Icier, 2009; Nicoli et al., 1999; Pashazadeh et al., 2020a; Zannou et al., 2021). Nonetheless, no statistical difference had been detected in the anthocyanin content of sweet cherries dried at 60, 70, and 80°C (Ouaabou et al., 2020). These findings suggested that not only the drying conditions can affect the antioxidant properties of the agricultural products but also the type and intrinsic characteristics of the raw material are determinants.

**TABLE 6:** Antioxidant properties of date plum dried in different drying conditions.

Temperature (°C)	Air velocity, m/s	Total phenolic, mg/g	Total flavonoid, mg/g	DPPH, mmol/g	FRAP, mmol/g
50	0.5	3.93 ± 0.43^c^	1.11 ± 0.15^d^	43.72 ± 2.46^a^	41.14 ± 1.68^b^
	1.0	2.49 ± 0.32^fe^	0.85 ± 0.11^gf^	29.37 ± 8.55^a^	41.86 ± 3.69 g
	1.5	3.50 ± 0.03^d^	1.27 ± 0.11^c^	37.01 ± 1.38^a^	43.66 ± 1.35^fedc^
60	0.5	1.47 ± 0.06 g	1.00 ± 0.11^fed^	31.76 ± 3.37^a^	39.81 ± 0.40^gf^
	1.0	2.24 ± 0.18^f^	0.99 ± 0.09^fed^	44.21 ± 3.41^a^	42.86 ± 0.80^b^
	1.5	2.66 ± 0.12^e^	1.47 ± 0.08^b^	39.45 ± 2.85^a^	43.52 ± 1.93^dcb^
70	0.5	4.57 ± 0.04^a^	1.37 ± 0.03^cb^	35.92 ± 2.50^a^	42.4 ± 1.62^fed^
	1.0	4.38 ± 0.18^ba^	1.66 ± 0.07^a^	54.03 ± 2.95^a^	42.99 ± 1.39^a^
	1.5	4.36 ± 0.39^cba^	1.03 ± 0.02^ed^	42.38 ± 0.43^a^	41.00 ± 1.24^cb^
80	0.5	3.43 ± 0.17^d^	0.68 ± 0.04^h^	42.97 ± 2.49^a^	42.73 ± 2.19^cb^
	1.0	4.12 ± 0.23^cb^	0.79 ± 0.07^hg^	38.61 ± 1.27^a^	44.85 ± 1.13^edcb^
	1.5	4.35 ± 0.22^cba^	0.89 ± 0.07^gfe^	32.54 ± 2.17^a^	42.8 ± 2.49^gfe^

*Note*: Data with the same superscript letters in a column are not significantly different (*p* > 0.05).

Abbreviations: DPPH, 2,2‐diphenyl‐1‐picrylhydrazyl; FRAP, ferric reducing antioxidant power.

### 3.6 Effects of drying on phenolic compounds

Thirteen phenolic compounds include gallic acid, catechin, chlorogenic, caffeic acid, vanillic acid, epicatechin, syringic, p‐coumaric acid, ferulic acid, sinapic acid, quercetin‐3‐glucoside, salicylic acid, and resveratrol were identified from date plum samples (Table 7). Previous studies have reported that date plum is a rich source of phenolic compounds such as gallic acid, vanillic acid, caffeic acid, ferulic acid, salicylic acid, quercetin, and ρ‐coumaric acid (Ayaz et al., 1997; H. Gao et al., 2014). In the present study, the phenolic compounds showed significant differences among drying conditions (*p* < 0.05). As can be seen in Table 7, the increase in drying temperature induced the increase of phenolic acid content, except for caffeic acid and ferulic acid. The values of gallic acid, catechin, chlorogenic acid, caffeic acid, vanillic acid, syringic acid, and p‐coumaric acid were lower in fresh than in dried fruit. On the other hand, the values of epicatechin, ferulic acid, sinapic acid, quercetin‐3‐glucoside, salicylic acid, and resveratrol were higher in fresh than in dried fruit (Table 7). The samples dried at 70°C had the highest values of gallic acid (341.79 mg/kg), catechin (65.80 mg/kg), chlorogenic acid (4.49 mg/kg), syringic acid (5.65 mg/kg), quercetin‐3‐glucoside (14.28 mg/kg), and resveratrol (2.26 mg/kg). Moreover, the highest values of vanillic acid (73.16 mg/kg), ferulic acid (8.66 mg/kg), and salicylic acid (13.75 mg/kg) were found at 60°C. In the same agreement with our findings, several studies have reported that the drying at the temperatures ranging from 60°C to 70°C preserve the most phenolic compounds (X. Gao et al., 1996; Erbay & Icier, 2009; Nicol et al., 1999; Ouaabou et al., 2020; Pashazadeh et al., 2020a; Zannou et al., 2021).

**TABLE 7:** Effect of drying conditions on the phenolic compounds date plum (mg/kg).

Temperature (°C)	Air velocity, m/s	Gallic acid	Catechin	Chlorogenic acid	Caffeic acid	Vanillic acid	Epicatechin	Syringic acid	*p* ‐Coumaric acid	Ferulic acid	Sinapic acid	Quercetin‐3‐glucoside	Salicylic acid	Resveratrol
Fresh Sample		169.18 ± 0.05	28 ± 0.23	0.00 ± 0.00	2.23 ± 0.01	52.68 ± 0.13	20.3 ± 0.34	2.65 ± 0.02	8.45 ± 0.21	8.2 ± 0.10	134.53 ± 0.34	21.25 ± 0.48	20.03 ± 0.25	3.125 ± 0.01
50	0.5	162.31 ± 8.50^f^	4.70 ± 0.51^f^	2.11 ± 0.02^cb^	7.61 ± 0.01^a^	53.93 ± 2.40^dc^	8.08 ± 0.35^dcb^	2.25 ± 0.10^g^	5.87 ± 0.74^g^	7.58 ± 0.33^ba^	13.80 ± 0.24^a^	3.96 ± 0.05^g^	8.55 ± 0.98^dc^	2.14 ± 0.01^edc^
	1.0	288.84 ± 8.69^c^	49.68 ± 6.67^cb^	2.13 ± 0.10^cb^	5.12 ± 0.05^c^	51.61 ± 0.04^dc^	7.56 ± 0.11^dcb^	3.83 ± 0.26^ed^	10.43 ± 0.22^fed^	7.67 ± 1.77^ba^	5.59 ± 1.22^dc^	6.54 ± 1.12^fe^	13.40 ± 1.78^b^	2.15 ± 0.02^c^
	1.5	259.97 ± 18.32^d^	47.97 ± 2.05^c^	2.30 ± 0.60^cb^	3.07 ± 0.21^f^	45.62 ± 8.79^ed^	7.38 ± 0.30^dcb^	3.48 ± 0.25^e^	9.14 ± 1.10^f^	6.39 ± 0.07^cb^	6.85 ± 0.87^c^	5.66 ± 0.03^f^	10.06 ± 0.53^c^	2.12 ± 0.00^ed^
60	0.5	292.11 ± 5.78^c^	50.25 ± 3.13^cb^	2.68 ± 0.26^b^	6.75 ± 0.30^b^	49.17 ± 0.06^dc^	10.52 ± 0.09^a^	4.16 ± 0.56^dc^	10.72 ± 1.40^edc^	7.64 ± 1.87^ba^	14.48 ± 0.13^a^	8.29 ± 0.15^d^	13.15 ± 0.33^b^	2.15 ± 0.0^c^
	1.0	276.68 ± 16.03^dc^	29.58 ± 4.20^d^	2.63 ± 0.18^b^	6.81 ± 0.22^b^	73.16 ± 0.83^a^	10.38 ± 0.29^a^	4.40 ± 0.22^c^	9.64 ± 0.12^ed^	8.66 ± 0.42^a^	14.70 ± 0.44^a^	7.86 ± 0.45^ed^	13.75 ± 1.17^b^	2.14 ± 0.01^edc^
	1.5	184.29 ± 10.47^f^	21.12 ± 0.57^e^	1.76 ± 0.01^c^	4.49 ± 0.19^d^	54.40 ± 0.87^dc^	8.70 ± 0.38^b^	5.65 ± 0.04^a^	5.30 ± 0.62^g^	6.45 ± 0.87^cb^	10.97 ± 2.31^b^	3.91 ± 0.28^g^	7.26 ± 1.42^ed^	2.12 ± 0.02^e^
70	0.5	341.79 ± 11.45^a^	60.54 ± 0.23^ba^	4.49 ± 0.01^a^	4.04 ± 0.13^e^	63.33 ± 2.47^b^	7.36 ± 0.05^dcb^	5.46 ± 0.11^ba^	12.14 ± 0.03^c^	6.73 ± 0.29^cba^	9.17 ± 0.93^b^	14.28 ± 0.43^a^	5.91 ± 0.65^fe^	2.26 ± 0.00^a^
	1.0	277.14 ± 15.05^dc^	65.80 ± 1.18^a^	2.35 ± 0.33^b^	4.64 ± 0.36^d^	48.85 ± 1.79^dc^	6.90 ± 0.75^dc^	4.24 ± 0.12^dc^	11.55 ± 0.74^dc^	6.49 ± 0.07^cb^	13.19 ± 0.29^a^	10.97 ± 0.35^c^	4.99 ± 0.20^gf^	2.26 ± 0.00^b^
	1.5	258.85 ± 1.57^d^	49.68 ± 6.29^cb^	2.54 ± 0.31^b^	3.09 ± 0.07^f^	44.58 ± 3.85^ed^	7.17 ± 0.69^dc^	4.00 ± 0.09^dc^	10.29 ± 0.54^fed^	5.07 ± 0.46^c^	4.35 ± 1.73^d^	8.94 ± 0.14^d^	3.32 ± 0.41^g^	2.19 ± 0.00^b^
80	0.5	314.75 ± 2.44^b^	48.60 ± 0.73^cb^	2.43 ± 0.09^b^	2.07 ± 0.01 g	50.57 ± 2.47^dc^	8.18 ± 0.15^cb^	2.92 ± 0.15^f^	19.75 ± 0.23^a^	6.41 ± 0.18^cb^	5.27 ± 0.20^dc^	11.28 ± 0.71^c^	10.42 ± 0.06^c^	2.12 ± 0.00^ed^
	1.0	342.23 ± 1.22^a^	56.47 ± 0.40^cb^	2.48 ± 0.02^b^	2.23 ± 0.05 g	41.15 ± 1.35^e^	5.81 ± 0.18^e^	4.99 ± 0.10^b^	14.73 ± 0.35^b^	6.35 ± 0.11^cb^	13.97 ± 0.89^a^	10.69 ± 0.09^c^	17.41 ± 1.01^a^	2.12 ± 0.00^ed^
	1.5	234.79 ± 3.74^e^	46.65 ± 4.83^c^	2.68 ± 0.04^b^	1.65 ± 0.02^h^	57.94 ± 1.98^cb^	6.65 ± 0.51^ed^	5.35 ± 0.03^ba^	13.96 ± 0.21^b^	7.36 ± 0.45^ba^	9.98 ± 0.06^b^	12.77 ± 1.58^b^	14.19 ± 0.85^b^	2.14 ± 0.01^dc^

*Note*: Different lowercase letters in the same column mean statistical difference at *p*≤ 0.05.

## 4 CONCLUSION

The present study attempted to examine the characterization of dried date plum and the influence of the drying process on its total phenolic compound, rehydration, microstructure, antioxidant activity, and phenolic compounds. The results showed that the convective drying of the date plum increased the total phenolic content, flavonoid content, antioxidant activity, and individual phenolic compounds when increasing the temperature up to 70°C. Additionally, the drying process showed significant effects on microstructure, drying, and rehydration kinetics. The best mathematical models of drying and rehydration kinetics were found to be Midilli and Weibull models, respectively. These findings have significant implications for understanding how to assess the drying parameters of rehydration, antioxidant activity, and phenolic compounds of date plum. This study revealed that the date plum is rich in bioactive ingredients, and the dried product can be used for multipurpose in food, cosmetic, and drug industries. Further research should focus on other drying methods to produce interesting findings on the best drying conditions for date plums.

## AUTHOR CONTRIBUTIONS

**Awadalgeed M. A. Hassan**: conceptualization; data curation; methodology; visualization; writing – original draft. **Oscar Zannou**: conceptualization; data curation; methodology; visualization; writing – original draft; writing – review & editing. **Hojjat Pashazadeh**: Conceptualization; Data curation; Methodology; Visualization; Writing – original draft; Writing – review & editing. **Ali Ali Redha**: data curation; funding acquisition; validation; visualization; writing – original draft; writing – review & editing. **Ilkay Koca**: conceptualization; data curation; funding acquisition; methodology; project administration; supervision; validation; visualization; writing – original draft; writing – review & editing.

## CONFLICT OF INTEREST

None to declare.
